# Determinants of ligand binding and catalytic activity in the myelin enzyme 2′,3′-cyclic nucleotide 3′-phosphodiesterase

**DOI:** 10.1038/srep16520

**Published:** 2015-11-13

**Authors:** Arne Raasakka, Matti Myllykoski, Saara Laulumaa, Mari Lehtimäki, Michael Härtlein, Martine Moulin, Inari Kursula, Petri Kursula

**Affiliations:** 1 Faculty of Biochemistry and Molecular Medicine, University of Oulu, Oulu, Finland; 2 Biocenter Oulu, University of Oulu, Oulu, Finland; 3 Department of Biomedicine, University of Bergen, Bergen, Norway; 4 Helmholtz Centre for Infection Research at German Electron Synchrotron (DESY), Hamburg, Germany; 5 European Spallation Source (ESS), Lund, Sweden; 6 Institut Laue-Langevin (ILL), Grenoble, France

## Abstract

2′,3′-cyclic nucleotide 3′-phosphodiesterase (CNPase) is an enzyme highly abundant in the central nervous system myelin of terrestrial vertebrates. The catalytic domain of CNPase belongs to the 2H phosphoesterase superfamily and catalyzes the hydrolysis of nucleoside 2′,3′-cyclic monophosphates to nucleoside 2′-monophosphates. The detailed reaction mechanism and the essential catalytic amino acids involved have been described earlier, but the roles of many amino acids in the vicinity of the active site have remained unknown. Here, several CNPase catalytic domain mutants were studied using enzyme kinetics assays, thermal stability experiments, and X-ray crystallography. Additionally, the crystal structure of a perdeuterated CNPase catalytic domain was refined at atomic resolution to obtain a detailed view of the active site and the catalytic mechanism. The results specify determinants of ligand binding and novel essential residues required for CNPase catalysis. For example, the aromatic side chains of Phe235 and Tyr168 are crucial for substrate binding, and Arg307 may affect active site electrostatics and regulate loop dynamics. The β5-α7 loop, unique for CNPase in the 2H phosphoesterase family, appears to have various functions in the CNPase reaction mechanism, from coordinating the nucleophilic water molecule to providing a binding pocket for the product and being involved in product release.

Myelin is a biologically unique cellular membrane structure, which envelops selected axonal segments in the nervous system and enables fast saltatory nerve impulse conduction and the correct functioning of the vertebrate nervous system^1^. A mature myelin sheath has two main morphological compartments. Its insulative nature is promoted by compact myelin, which is formed of tightly stacked lipid bilayers held together by a group of highly abundant myelin proteins^2^. Non-compact myelin has an important role in myelin maintenance, higher water content, and a different protein composition^2^,^3^.

2′,3′-cyclic nucleotide 3′-phosphodiesterase (CNPase) comprises 4% of total myelin protein in the central nervous system (CNS), being the most abundant CNS non-compact myelin protein^4^. CNPase is expressed as two isoforms through alternative splicing^5^,^6^. Both isoforms are abundantly expressed within the cytoplasmic compartment of non-compact myelin, while low levels of the isoform 2 mRNA are also detected in tissues outside the nervous system^7^,^8^. Isoform 2 is able to undergo mitochondrial import mediated by its N-terminal import sequence^9^. CNPase consists of an N-terminal polynucleotide kinase-like domain^10^, a catalytic phosphodiesterase domain^11^, and a membrane-anchored C-terminal tail, which contains a cysteine residue that undergoes isoprenylation^12^,^13^,^14^. CNPase is a potential autoantigen in multiple sclerosis^15^, and variability in CNPase expression levels has been linked to neurological and psychiatric disorders, including Alzheimer’s disease, Down syndrome^16^, schizophrenia^17^,^18^,^19^, and schizophrenia-related catatonic depression^20^. CNPase-deficient mice develop axonal swelling and degeneration, which further leads to progressive motor deficiencies and premature death^21^. In these mice, the inner tongue of myelin is most notably deformed, although myelin appears morphologically normal^22^.

The biological role of CNPase is unclear at the moment. CNPase might be involved in several cellular processes, since it interacts with RNA^23^,^24^, calmodulin^24^,^25^, and the cytoskeleton^26^,^27^. The significance of the latter in oligodendrocyte process outgrowth has been demonstrated in cell culture studies^27^. CNPase has also been shown to inhibit translation^23^, to modulate mitochondrial membrane permeability^28^, to inhibit the replication of several viruses^29^,^30^, and to have putative ATP/GTPase activity^31^. Additionally, CNPase can rescue yeast deficient in tRNA splicing *in vivo*, when the 2H domain of yeast tRNA ligase has been inactivated^32^. The structure, function, and significance of CNPase have recently been reviewed^33^.

The C-terminal phosphodiesterase domain is the best characterized domain of CNPase. This domain is highly conserved in mammalian CNPases^34^, and structurally and functionally, it belongs to the 2H phosphoesterase superfamily^11^,^35^,^36^, which includes several clades of enzymes involved in nucleotide or RNA metabolism^37^. The catalytic domain enables the rapid hydrolysis of nucleoside 2′,3′-cyclic phosphates to nucleoside 2′-phosphates^34^,^38^. The presence of endogenous adenosine 2′,3′-cyclic monophosphate (2′,3′-cAMP) was recently described in astrocytes, microglia^39^, and oligodendrocytes^40^. 2′,3′-cAMP has putative biological functions in mitochondrial Ca^2+^ release^28^ and A_2A_ adenosine receptor-mediated TNF-α inhibition^41^, and enzymatic 2′,3′-cAMP depletion by CNPase upon traumatic brain injury has been suggested^42^,^43^.

We previously focused on the essential catalytic residues and the phosphodiesterase reaction mechanism of CNPase^34^. Here, using a wide panel of mutations, not all directly affecting the catalytic residues, we investigated the active site of mouse CNPase catalytic domain (CNPcat) by biochemical and structural methods. Additionally, we characterized the perdeuterated CNPase catalytic domain (dCNPcat) and determined its crystal structure at 1.04-Å resolution, allowing the observation of active site protonation states and dynamics, as well as water networks. Altogether, our results provide a more detailed picture of the structure-function relationships in CNPase and shed light on the roles of several active-site residues involved in either direct or indirect ligand binding. The results allow to further understand substrate binding and specificity determinants in 2H phosphoesterases.

## Results

### The CNPase active site and its surroundings

**Figure 1:**
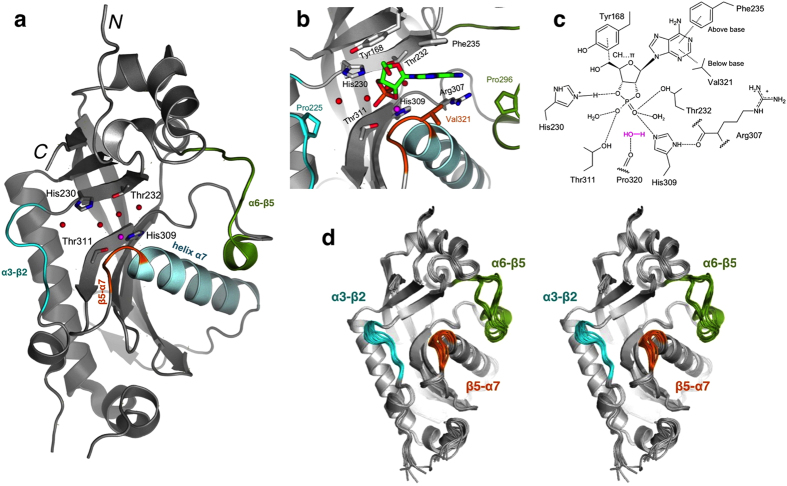
The CNPase catalytic domain active site and essential ligand interactions. (**a**) Mouse CNPase catalytic domain with the active site 2H phosphoesterase family-signature His-x-Thr-x drawn as cylinders. Helix α7 (light blue), unique to CNPase, as well as the dynamic α3-β2 (cyan), α6-β5 (dark green), and β5-α7 (orange) loops are highlighted. Additionally, the active-site water molecules are shown in red and the nucleophilic water in magenta. (**b**) A detailed view of the active site, with residues of interest to the current study indicated. Substrate (2′,3′-cAMP, green) is in the active site, based on its coordinates in the H309S mutant complex structure. (**c**) Schematic illustration of the binding interactions of a substrate within the active site. (**d**) Stereo image of a superposition of all mutant structures obtained in this study, demonstrating loop flexibility in CNPcat.

To justify targets for mutagenesis, earlier knowledge on CNPase structure and activity was employed. The CNPase catalytic site, lying in a groove between two lobes, has pseudo two-fold symmetry and contains two apposing His-X-Thr-X (X denotes a hydrophobic residue) motifs, characteristic for 2H phosphoesterases (Fig. 1a)^11^,^35^,^37^. Four water molecules at the bottom of the active site also obey the two-fold symmetry^36^,^38^. Upon substrate binding, the His and Thr residues, together with the water molecules, coordinate the 2′,3′-cyclic monophosphate group^34^. In mouse CNPase, this is done by His230, Thr232, His309, and Thr311, in a manner, where a nucleophilic water molecule activated by His309 can attack the cyclic phosphate and a pentavalent transition state is formed. His230 then donates a proton to the ribose 3′-O atom, which acts as the leaving group, and a 2′-phosphate product is formed^34^. In addition to hydrogen bonds to the His-X-Thr-X motifs and water molecules, the binding of ligands is mediated by two means (Fig. 1b,c): (1) the aromatic base of the nucleotide is sandwiched between the side chains of Phe235 and Val321, and (2) the ribose moiety forms C-H…π hydrogen bonds to the aromatic side chain of Tyr168^38^. The active-site cleft is also able to fit larger ligands, such as nicotinamide adenine dinucleotide phosphate (NADP^+^), and possibly even oligonucleotides^34^.

The cleft is flanked by two proline-containing loops (denoted α3-β2 and α6-β5, see Fig. 1a,d), and the β5-α7 loop in front of the active site core has a role during catalysis^34^. The β5-α7 loop leads into helix α7, which is conserved in vertebrate CNPases, but absent in other 2H phosphoesterases^36^. This helix may be functionally important in determining CNPase stereospecificity^44^. In the vicinity of the bound nucleotide and the catalytic core, the side chain of Arg307 can swing towards and away from the active site and, therefore, could play a role in the catalytic cycle^38^.

The loops surrounding the active site could affect substrate binding and catalysis. Figure 1d presents a superposition of all CNPcat structures determined in this study (see below), showing flexibility that may be crucial to activity-related dynamics in CNPase.

### Initial characterization of CNPase catalytic domain variants

In order to investigate the ligand binding properties of CNPcat, we expressed and purified CNPcat with the following mutations: H230Q, H309Q, H230S, H309S, T232A, T311A, F235A, F235L, V321A, R307Q, Y168A, Y168S, P225G, P296G, and H230Q/H309Q. In all cases, the proteins were straightforward to produce and behaved similarly to the wild-type protein during purification. The presence of the mutations was confirmed using DNA sequencing, mass spectrometry, and X-ray crystallography (Supplementary Table S1, Supplementary Table S2).

Mass spectrometry was used to examine hydrogen-deuterium exchange in CNPcat and dCNPcat (Supplementary Table S3). Comparing the measured mass of dCNPcat in D_2_O (25976.0 Da) with the theoretical mass of 100% perdeuterated protein (26012.1 Da), the perdeuteration degree of dCNPcat was very high (97.9%). Additionally, the amount of exchanging protons and deuterons was in the same range for both samples (Supplementary Table S3), but not identical, indicating a small chemical difference between protons and deuterons.

Small-angle X-ray scattering (SAXS) and synchrotron radiation circular dichroism spectroscopy (SRCD) were used for quality control of CNPcat variant structure in solution (Supplementary Fig. S1, Supplementary Table S4). All mutants were folded, and in all cases, the radius of gyration, the maximum dimension, and the secondary structure content were similar to the wild-type protein. Hence, the point mutations did not significantly alter CNPcat folding. Additionally, thermal stability of dCNPcat (T_m_ = 57 °C, Supplementary Fig. S1) was similar to the hydrogenated protein^34^,^45^.

### Essential catalytic residues

We next investigated the effect of mutations on catalysis (Table 1). In addition to activity assays, we crystallized all mutants and determined crystal structures with different active-site ligands (Supplementary Table S1).

**Figure 2:**
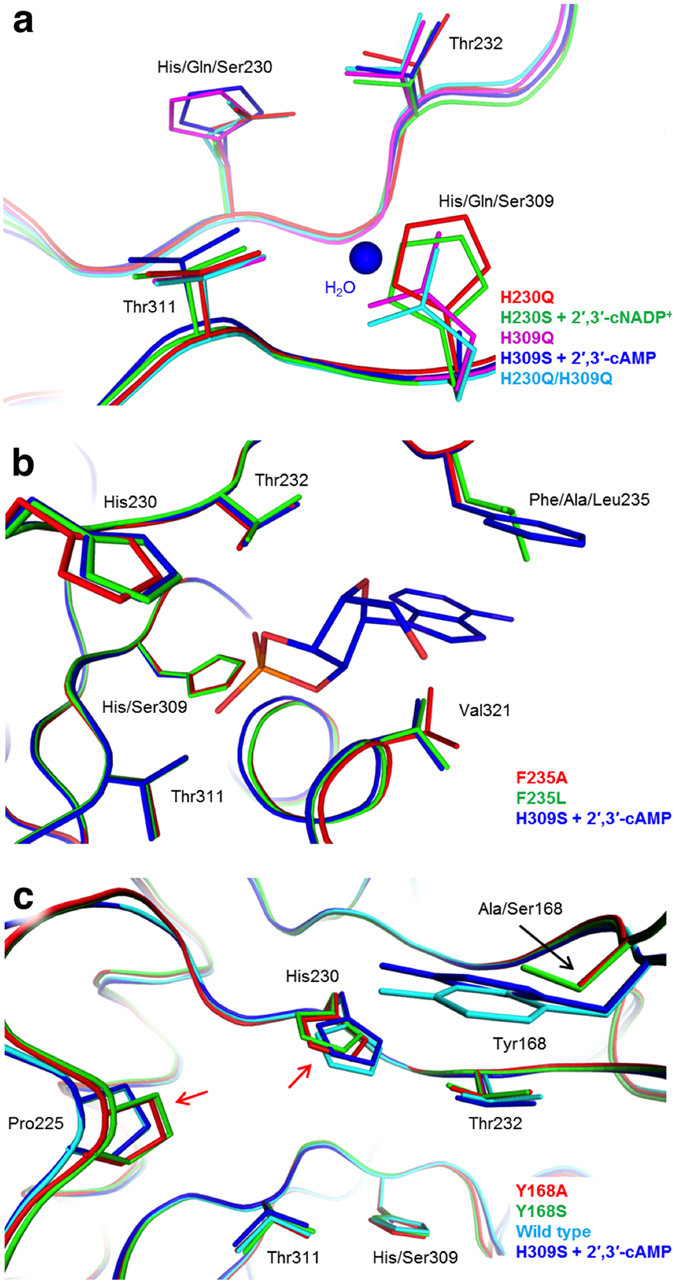
Inactivating mutations. (**a**) Superposition of all His230 and His309 mutant structures shows minimal molecular rearrangement within the active site. The additional water molecule that partially substitutes for the absent His309 imidazole in the H309S substrate complex structure is indicated (blue). (**b**) Superposition of Phe235 mutants with the H309S substrate complex demonstrates minimal molecular rearrangement in the active site, despite almost complete inactivation. (**c**) Mutations in Tyr168 result in minor molecular rearrangements in the active site, most notably at position 168, as well as at Pro225 and His230.

Mutation of active-site histidine residues (H230Q, H230S, H309Q, H309S, and H230Q/H309Q double mutant) abolishes CNPase activity^34^. Overall, the structures of H230S, H309S^34^, H230Q, H309Q, and H230Q/H309Q are highly similar (Fig. 2a). However, only H230S and H309S could be used to trap nucleotide ligands within the active site, whereas H309Q and the H230Q/H309Q double mutant could not^34^. In H309S, an extra water molecule resides at the position of the missing imidazole ring, aiding in achieving correct binding coordination^34^.

In addition to the catalytic His mutants, near-complete catalytic inactivation is evident also in T232A, T311A, F235A, F235L, and Y168A (Table 1). The inactivation by the T232A and T311A mutations was expected^35^: threonine or serine is found in the catalytic motif in all 2H phosphoesterases, and in CNPcat, they are involved in all stages of catalysis, in achieving the correct substrate binding mode together with His230, His309, and the active-site water network. The catalytic activity in the case of T232A may not be completely lost, as T232A was crystallized in the presence of 2′,3′-cAMP and excellent density for a reaction product is visible in the active site (Supplementary Fig. S2a). The time scale of crystallization and crystal storage far exceeds the slow enzymatic activity of the mutant.

Together with Val321, Phe235 has a direct role in substrate binding^34^,^38^,^46^. Both F235A and F235L mutations decrease k_cat_, although Phe235 is not directly involved in the enzymatic reaction. While recognizing the caveat that the high K_M_ for these mutants makes k_cat_ rather inaccurate, this implies that the π-π stacking between Phe235 and the nucleotide base is not only required for substrate affinity, but it also may define correct substrate docking into a productive binding mode (Fig. 2b). Unproductive binding modes may lead to false interpretations of reaction kinetics^47^. In the F235L structure, the flexible active site β5-α7 loop (amino acids 319 – 321, see Fig. 1) is in the closed conformation. This loop is often found in a mixed conformation, and its conformation depends on the reaction step. The functional relevance of the β5-α7 loop will be discussed below.

Tyr168 is one of the aromatic residues on the CNPcat surface that we suggested to form a binding surface for RNA^34^. Tyr168 interacts with the substrate ribose moiety through C-H…π hydrogen bonding^38^ (Fig. 1b,c). We carried out mutagenesis to study the role of the aromatic side chain of Tyr168. While the Y168A mutation decreases catalytic activity, Y168S retains it, albeit with an elevated K_M_ compared to the wild-type enzyme. An increased K_M_, whilst retaining similar k_cat_, is an indication of decreased substrate binding affinity, with a minimal effect on actual catalysis. In the absence of the aromatic ring, the serine OH group appears to be enough to retain catalytic activity; this could be related to differences in water networks or overall dynamics in Y168A and Y168S. To further investigate the binding mode, we also attempted co-crystallization of CNPcat Y168S in the presence of 2′,3′-cAMP, but even residual ligand density in the active site could not be observed. In the mutants, the missing ion-dipole interaction between the Tyr168 hydroxyl group and the His230 imidazole ring apparently causes His230 and Ala/Ser168 to reposition in the active site by ~1 Å (Fig. 2c). Additionally, loop α3-β2 moves towards the active site by the same distance. These structural features suggest that Tyr168 also has a role in fine-tuning the active site conformation with its bulkiness.

### Active-site dynamics

**Figure 3:**
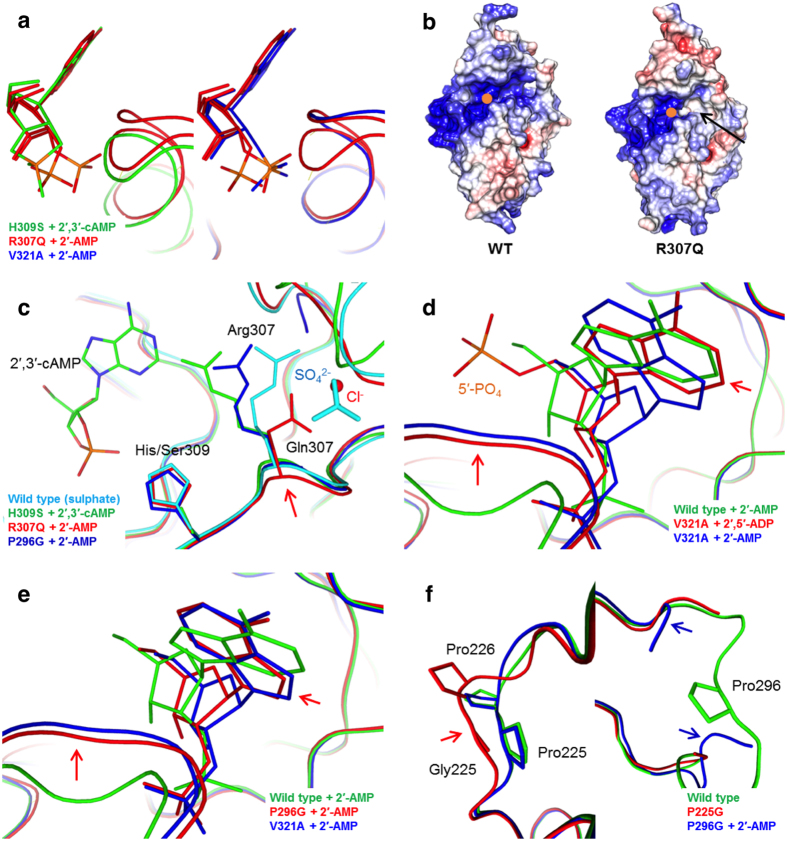
Several mutants highlight dynamics within the active site. (**a**) Superposition of R307Q with the H309S substrate complex and the V321A product complex demonstrate that in R307Q, the product 2′-phosphate can adopt different conformations, linked to the β5-α7 loop conformation. (**b**) Comparison of the surface electrostatics between wild-type CNPcat and R307Q reveals changes in the immediate proximity of the active site (arrow), caused by the absence of the positively charged Arg307. The catalytic site has been indicated with an orange dot. (**c**) The conformations of Arg307 in the sulphate complex and Gln307 in R307Q suggest that Arg307 has a dynamic role, which is possibly related to the adjacent anion-binding pocket. In these two structures, this pocket is occupied by sulphate (cyan) and chloride (red), respectively. Conformational variability of Arg307 is evident also in the P296G structure. For clarity, only the substrate from the H309S structure is shown in the active site. (**d**) The presence of the 5′-phosphate causes the ligand to retract slightly from the active site in V321A, but the β5-α7 loop remains open and the 2′-phosphate is bound under it (arrow). (**e**) In P296G, the β5-α7 loop adopts the open conformation, similarly to the V321A structures, showing that loop opening is not caused by the absence of Val321. (**f**) Mutations from Pro to Gly at positions 225 and 296 affect loops α3-β2 and α6-β5, respectively. Loop α6-β5 is partially disordered in many structures.

The complex with adenosine 2′-monophosphate (2′-AMP) reveals that the R307Q mutant binds the product in a mixed conformation, whereby only the phosphate position changes, and the β5-α7 loop also presents a linked double open/closed conformation (Supplementary Fig. S2b, Fig. 3a). Also, similarly to V321A^34^, R307Q presents an elevated K_M_, while k_cat_ remains at the wild-type enzyme level. While Arg307 can be assumed to mainly affect ligand binding affinity, its mode of action is not entirely clear, as Arg307 makes no direct interactions with active site ligands in crystal structures. Arg307 may be important for active-site electrostatics (Fig. 3b), likely playing a role in attracting substrates to bind. Interestingly, Arg307 can have different conformations in crystal structures. In the substrate complex^34^, Arg307 is positioned towards the active site, like in the wild-type CNPcat apo structure^38^, whereas in R307Q, Gln307 is turned away – quite similarly to Arg307 in P296G complexed with 2′-AMP (see next paragraph), and notably, in the sulphate complex determined earlier^38^, where wild-type CNPcat coordinates a sulphate ion at the nearby anion binding pocket (Fig. 3c). Gln307 similarly coordinates a chloride ion in the anion binding pocket, together with Trp289 and the backbone amide of Gly305. In all structures, the 2.8-Å hydrogen bond between His230 Nδ1 and the carbonyl O of residue 307 is retained, which results in a ~1 Å retraction of the main chain at position 307 in the sulphate complex^38^ and R307Q (Fig. 3c). In many structures, the side chain of Arg307 forms hydrogen bonds to a main-chain carbonyl group at the N-terminal end of helix α7, suggesting its conformation may be linked to the opening and closing of the β5-α7 loop.

The structure of the CNPcat H230S mutant complexed with NADP^+^ 34 demonstrates that in addition to the reactive phosphate, the dinucleotide is predominantly bound *via* its adenine and nicotinamide bases and stabilized through the ribose moieties, whereas the pyrophosphate linker does not directly interact with the enzyme. We previously determined a V321A structure in complex with 2′-AMP^34^, in which the β5-α7 loop was open. While we have also obtained structures with an open β5-α7 loop from other mutants and the wild-type protein in complex with products, this has been particularly easy with V321A. This could relate to a higher binding affinity for the product, which would result in enzyme inhibition *via* hindered product release, and may explain the increased K_M_ value^34^. To further investigate the relevance of ligand size, we crystallized V321A in the presence of adenosine 2′,5′-bisphosphate (2′,5′-ADP), which, in addition to the 2′-phosphate, also contains a 5′-phosphate, thus mimicking the first 5′-phosphate of an RNA molecule. We obtained structures in the space groups P2_1_ and P2_1_2_1_2_1_, and both structures allowed us to analyze the ligand orientation in its entirety (Supplementary Fig. S2c). Similarly to V321A in complex with 2′-AMP^34^, the 2′,5′-ADP complexes show the β5-α7 loop the in the open conformation, with direct interactions between the 2´-phosphate and the N terminus of helix α7 (Fig. 3d). The 5′-phosphate is visible and extends out of the active site towards bulk solvent. In an RNA oligonucleotide, the 5′-phosphate will be followed by another ribose and a base; several aromatic residues on the CNPase surface are in position to interact with these groups^38^.

Interestingly, the mutant P296G has a marginally higher k_cat_ than the wild-type enzyme (Table 1). In the P296G structure, a 2′-AMP product is present in the active site (Supplementary Fig. S2d). In contrast to the wild type enzyme, the adenine base is in a conformation similar to that seen in V321A before, and the β5-α7 loop has similarly adopted the open state (Fig. 3e). The latter, together with earlier liganded structures with an open β5-α7 loop^34^, suggests that loop opening is not an artifact caused by the V321A mutation, but rather a stabilized state so far not seen in crystal structures of the wild-type protein in complex with 2′-AMP. Additionally, the activity is probably not hindered by the observed base-flipping phenomenon, as demonstrated by P296G. Furthermore, as mentioned above, Arg307 has moved slightly away from the active site to the general direction where Gln307 would reside in the R307Q structure and Arg307 in the sulphate complex^38^ (Fig. 3c). This, again, suggests that active site loop dynamics are linked to the conformation of Arg307.

### Distal proline-containing loops

Pro225 and Pro296 reside in loops α3-β2 and α6-β5, respectively, flanking the edges of the active site – Pro296 near the adenine moiety and Pro225 on the 5′-side of the ligand, next to Pro226 (Fig. 1b). The k_cat_ values of P225G and P296G are slightly higher compared to the wild type enzyme (Table 1), most likely due to conformational flexibility offered by the glycine residues. P225G is the only mutant with an increased specificity constant (k_cat_/K_M_). We attempted to co-crystallize P225G in the presence of ligands. However, the reasonably low resolution (2.70 Å) crystal structure, which we obtained from crystals grown in the presence of reduced nicotinamide adenine dinucleotide 2′,3′-cyclic phosphate (2′,3′-cNADP^+^), contains only poorly defined ligand density in the active site (Supplementary Fig. S2d). In P296G, which exhibits a higher k_cat_ than wild-type CNPcat, while k_cat_/K_M_ remains in similar range, the mutation-containing loop α6-β5 is disordered in the crystal structure, but adopts a different conformation compared to wild-type CNPcat (Fig. 3f).

### Thermal stability and ligand binding

In order to investigate the ligand binding properties of CNPcat, we screened several ligands with differences in functional groups, measuring thermal stability midpoints (T_m_), or “melting points”, of wild-type CNPcat and the mutants (Supplementary Fig. S3). The results indicate that all the variants were correctly folded and heat-stable, with the largest destabilization, 8–9 °C, caused by the H230Q/H309Q double mutation. The catalytic motif, thus, is also important for CNPase stability. On the other hand, some mutant variants, such as P296G, even appeared slightly more stable than the wild-type protein.

**Figure 4:**
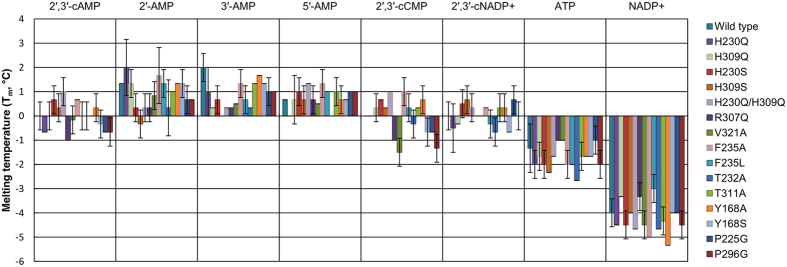
CNPcat thermal stability differences with ligand additives. The T_m_ values have been normalized to the T_m_ of each mutant in the absence of ligands.

Many of the ligands used in this study are known to bind CNPcat either from structures, enzyme activity assays^34^,^48^,^49^, or binding assays^35^. The CNPase mutants presented differences in T_m_ values with and without ligands, which included ones with variation in the monophosphate position (2′-AMP, 3′-AMP, 5′-AMP), ones with a hydrolysable cyclic phosphate (2′,3′-cAMP, cytidine 2′,3′-monophosphate (2′,3′-CMP), 2′,3′-cNADP^+^), the product from activity assays (NADP^+^), and adenosine 5′-triphosphate (ATP) (Table 1, Fig. 4, Supplementary Fig. S3). The ligands mostly present the same trends in T_m_ for all protein variants (Fig. 4). Nearly all variants are slightly stabilized by all the acyclic adenosine monophosphates.

While for the substrates 2′,3′-cAMP, 2′,3′-cCMP, and 2′,3′-cNADP^+^, the T_m_ values remain unchanged, differences are seen for reaction products. For NADP^+^, the T_m_ is generally much lower than for 2′-AMP. NADP^+^ has a much larger interaction area with the enzyme between the two lobes^34^, and it destabilizes the fold to some extent. Additionally, ATP lowers the thermal stability of CNPcat. The binding mode of ATP is not known, but the data are indicative of an interaction. While thermal stability assays are a fast and easy way to assess qualitative differences in ligand binding, they should be interpreted with caution.

### Deeper insights into catalysis from atomic-resolution crystallography

We prepared a perdeuterated variant of CNPcat and performed activity assays for perdeuterated and hydrogenated CNPcat with both H_2_O and D_2_O as reaction solvents. In H_2_O, dCNPcat exhibits similar k_cat_ and K_M_ to CNPcat. The k_cat_/K_M_ is marginally higher, indicating that the enzyme is functioning slightly more specifically (Table 1). Changing the reaction solvent to D_2_O has a more profound effect. Here, k_cat_/K_M_ remains similar, but both k_cat_ and K_M_ drop to approximately one third compared to the enzymes in H_2_O. Thus, dCNPcat remains chemically and functionally very similar to hydrogenated CNPcat, and the solvent effect has a much higher impact on catalysis than the isotope effect arising from perdeuteration. In D_2_O, the rapidly exchanging protons in the active site exchange to deuterons, and instead of regular hydrogen bonding, the ligands occupied in the active site experience stronger deuterium-mediated hydrogen bonds; in addition, reaction steps involving (de)protonation will surely be affected. K_M_ indicates that the affinity towards substrate is higher, whereas k_cat_ suggests that the rate-limiting step of catalysis is slower with deuterated reactive groups. To what extent each of these reflect the role of deuterated solvent, substrate/product, and protein, remains to be studied.

As the thin crystals of dCNPcat could not be grown to dimensions suitable for neutron diffraction, the structure was solved using X-ray crystallography and refined to an atomic resolution of 1.04 Å. This is by far the highest resolution obtained for CNPase so far. Recently, a poorly characterized 2H family member, U6 snRNA biogenesis 1 protein, was refined at 1.1-Å resolution^50^.

**Figure 5:**
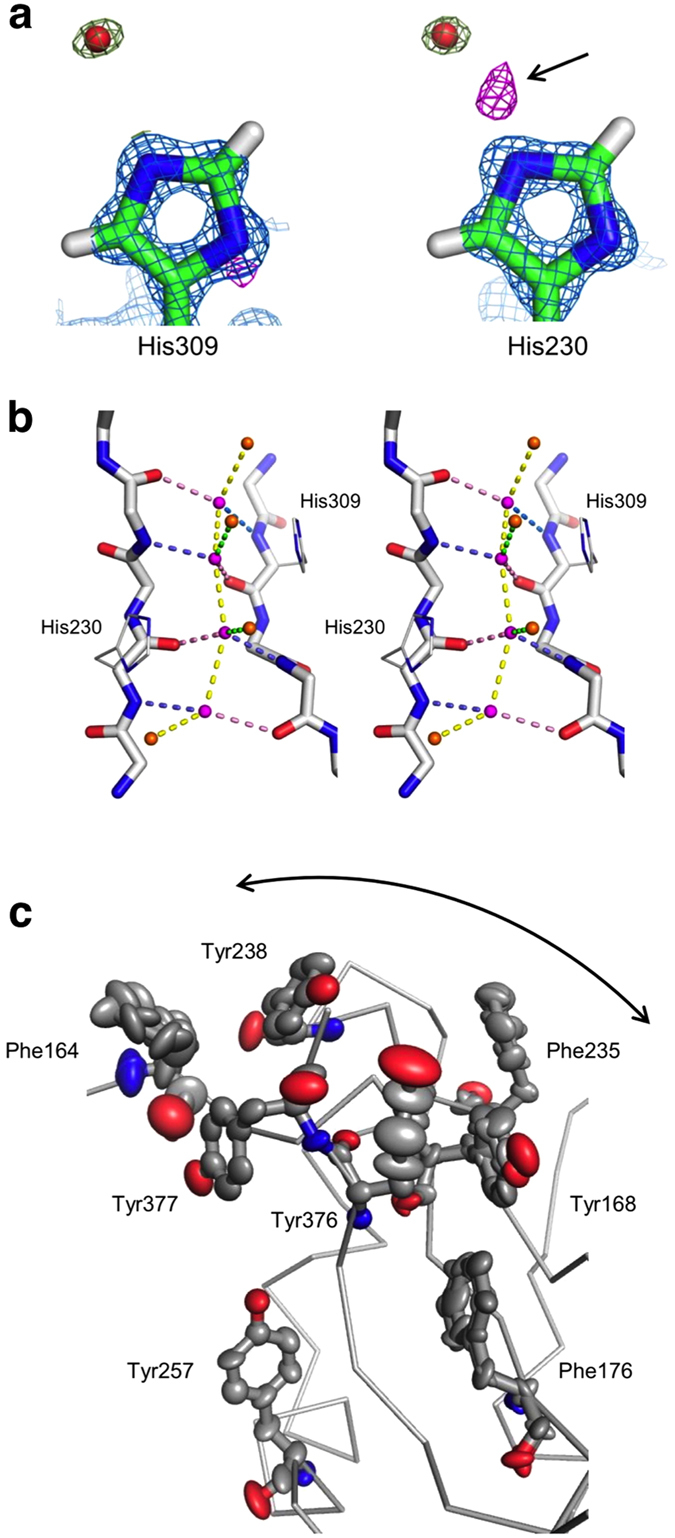
Atomic-resolution details of CNPcat. (**a**) 2F_o_-F_c_ electron density for His230, His309, and the proximal water molecules is shown at 3σ. Density for an extra proton of His230 is visible in F_o_-F_c_ maps contoured at 2.5 σ (purple). (**b**) Stereo image of the dCNPcat active site water network in the apo state. The hydrogen bonds are coloured as follows: backbone amide donating a hydrogen bond to water, blue; water donating a hydrogen bond to a backbone carbonyl, pink; hydrogen bonds that will bind the substrate cyclic phosphate, green; other hydrogen bonds between water molecules, yellow. (**c**) Anisotropic refinement reveals directional disorder of aromatic residues in the active site (at the right in this view) neighbourhood.

The proposed catalytic mechanism for CNPase involves two histidine residues in the active site; His309 deprotonates and activates the nucleophilic water molecule, and His230 coordinates the leaving group and protonates it upon completion of the reaction^34^. Examination of the electron density maps for these residues confirms that in the apo state, His309 is deprotonated and His230 protonated (Fig. 5a). The result is well in line with the proposed mechanism, as well as the CNPase pH optimum value of 6^51^.

The conserved water molecules at the bottom of the active site are well-defined in electron density. We observed difference electron density maps to detect locations of deuterium atoms, as at this resolution, for well-defined parts of the structure, they should be detectable for regions with low temperature factors. The water molecules in the active site are mobile enough to mask much of the difference density, but the surrounding chemical environment could be used in addition to define their orientations. The chain of 4 water molecules at the bottom of the active site is coupled to another group of 4 water molecules above them – representing a true apo active site, without any bound buffer components (Fig. 5b). The outcome of a more detailed analysis is surprising; out of the 4 water molecules at the bottom, the central ones have 5 coordination contacts, while both of the distal ones only have 4, in a classical tetrahedral setting. This indicates that the two middle water molecules are possibly forming bifurcated, or three-centered, hydrogen bonds and/or may change their orientation upon ligand binding^52^. We expect both of them to be hydrogen bond donors when substrate binds.

As the active site of dCNPcat is empty in the crystal structure, we determined an additional structure of CNPcat with no ligand bound; this was successful when using the F235L mutant. All earlier structures have included either nucleotides or buffer components. The positions of the active-site water molecules, as well as side chain conformations of catalytic residues were nearly identical in dCNPcat and F235L, suggesting any changes in activity of dCNPcat are related to the replacement of H by D in the protein, solvent, and exchangeable sites of the substrate. Attempts to co-crystallize ligands within the active site of dCNPcat failed (unpublished data).

The highest resolutions obtained previously for CNPase were slightly better than 2.0 Å; hence, anisotropic refinement has not been employed, and details of local disorder/flexibility have remained unrevealed. Anisotropic B factor refinement and alternative conformations reveal directional disorder in the vicinity of the catalytic cavity, for example concerning a number of aromatic residues (Fig. 5c). The disorder was partly built as alternative conformations, and partly dealt with by anisotropic refinement alone, depending on local electron density. The α3-β2 loop also exhibits a double conformation, showing it is flexible even in the crystal state. Residues with concerted disorder are likely to be involved, for example, in binding larger substrates, such as RNA.

At atomic resolution, it is not unusual to encounter side chain conformations violating geometric restraints. In the case of dCNPcat, the most drastic geometric outlier is Trp289, which is sandwiched through C-H…π interactions between Pro290 and Gly305. The side chain is bent, such that Cβ lies far from the plane of the aromatic ring. This feature is unambiguously defined in electron density. Such a conformation implies strain in the structure, and could be related to *e.g.* the anion binding pocket or the conformation of loop α6-β5 in the crystal structure. Overall, the atomic-resolution details on CNPase and its active site provide unprecedented detail on the structure-function relationships in 2H family enzymes.

## Discussion

We characterized an array of CNPcat mutants using structural and biochemical approaches. Our results verify the roles of assisting amino acids in the phosphodiesterase active site and uncover crucial roles for Tyr168 and Phe235, which are required for correct substrate binding prior to catalysis.

**Figure 6:**
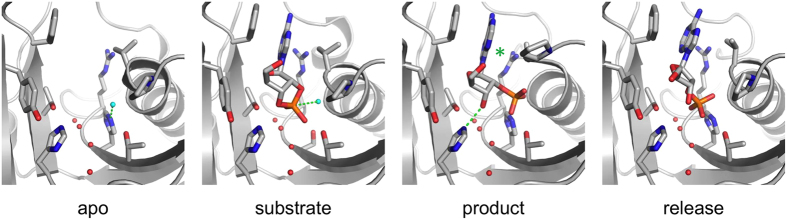
The CNPase reaction cycle. In the apo state, the β5-α7 loop is closed when a substrate enters. During catalysis, the product phosphate moiety flips into a cavity formed by the opening of the loop. The loop closes again, which results in product repositioning and release. The dashed lines represent the reaction step taking place in each state (from left to right: catalytic water activation by His309; nucleophilic attack to cyclic phosphate; protonation of ribose 3′-O by His230). Note the subtle shift in Arg307 orientation during catalysis and loop movement (green asterisk).

Active-site loops and Arg307 may function in regulating ligand binding through intramolecular dynamics. Taking our structural data together, we believe to have trapped distinct steps of the CNPase catalytic cycle (Fig. 6). Our observations allow us to speculate on active-site dynamics that take place during and after catalysis: The β5-α7 loop in both dCNPcat and F235L is closed, confirming the closed state of the active site in the apo form. In this state, Arg307 is situated close to the active site, and will remain so during the entry of a substrate. Initially, the substrate is locked in place through a hydrogen bonding network^34^, which allows the catalytic water molecule to be coordinated in a productive manner. During catalysis, the β5-α7 loop opens to expose the N terminus of helix α7 and to provide space for the product, which still remains locked in a very similar orientation as the original substrate. This loop opening might be modulated by the conformation of Arg307, since our R307Q structure demonstrates that in the absence of Arg307, the loop apparently has a much higher degree of freedom and adopts a mixed open/closed conformation. Here, the product bound to the active site also adopts two conformations; when the loop is closed, the position is comparable to the pre-catalytic cyclic phosphate, and when open, the phosphate resides in the pocket under the β5-α7 loop (Fig. 3a). After catalysis, the loop remains in an open state until it collapses, possibly in an Arg307-regulated manner. This state with the closed loop and bound product we observed before with the wild-type protein^38^; the conformation is rather different from the substrate and the product before loop collapse (Fig. 3e). The weakened hydrogen bonding between His230 and the ribose 3′-OH in this state could contribute to product release. Details of the putative Arg307 conformational change remain unclear. Similarly, the free energy barriers and the microscopic rate constants of each step, as well as the rate limiting step, remain undetermined at this time. Computational approaches are being planned to obtain more fine details on the CNPase catalytic cycle.

A conformational interplay between the β5-α7 loop and Arg307 is possible, and slight modulation of the active site coordination by conformational changes suggests that Arg307 could also otherwise be linked to active-site dynamics. For example, Arg307 could act as an affinity switch in substrate binding or product release through allosteric modulation by other factors, such as a ligand binding to the anion binding pocket. Our structural data indeed suggest a possibility to bind different anionic species to this pocket. Further characterization of the specificity of the anion binding pocket and its potential role in modulating the active site should also be undertaken.

The structural aspects discussed here provide new information about CNPase, which is a conserved 2H phosphoesterase in its own clade and acts differently from several other members of the 2H superfamily. Yet, it is also important in the context of other 2H phosphoesterases to understand the CNPase catalytic cycle, not only in a mechanistic view, but in a more spatial-dynamic manner. CNPase is structurally unique with its helix α7, which could be involved in stereospecificity and determination of substrate size and allows CNPases to function as 2′,3′-cyclic phosphodiesterases, whereas other 2H phosphoesterases, like plant CPDases, can additionally hydrolyze 1″,2″-cyclic ADP-riboses or are active on RNA substrates^37^. So far, we have only focused on adenine as a nucleotide base; for example, several modified bases that extend beyond the size of adenine have been described in tRNA molecules^53^. The destabilizing effect observed with ATP and NADP^+^ compared to 2′-AMP is interesting, and incites to study even longer ligands, such as oligonucleotides with terminal 2′,3′-cyclic or 2′-phosphates, which are intermediate molecules in tRNA processing^32^,^54^,^55^ and can potentially function as endogenous RNA-like substrates for CNPase. To conclude, our results provide novel information about the ligand binding properties and dynamics of CNPase and evoke new future research directions for this peculiar enzyme and its homologues in the 2H family.

## Methods

### Cloning, mutagenesis, and protein production

Mutations were generated to CNPcat (amino acids 179 – 398) in the pTH27 expression vector^45^,^56^, using the QuikChange II site-directed mutagenesis kit (Agilent Technologies). The mutations were verified by DNA sequencing. The proteins were expressed using auto-induction and purified as described earlier^45^,^57^. Briefly, the proteins were expressed in *Escherichia coli* Rosetta(DE3) and purified using Ni-NTA affinity chromatography, followed by proteolytic His-tag cleavage^58^ and size-exclusion chromatography.

For producing dCNPcat, the CNPcat cDNA was subcloned into the pETNKI-his3C-LIC-Kan vector^59^. Protein production was carried out at the deuteration laboratory of the Partnership in Structural Biology in Grenoble (France), essentially as described^60^. dCNPcat was expressed in *E. coli* Rosetta (DE3) cells in minimal medium, with glycerol-d_8_ as the carbon source. Cells were adapted from fully hydrogenated to fully deuterated medium in six steps of 1:10 inoculation before cultivation and protein expression at +30 °C in a Labfors fermenter (Infors). The purification of dCNPcat was performed similarly to CNPcat. The protein purified in H_2_O buffers was exchanged back to deuterium oxide (D_2_O) by centrifugal ultrafiltration, whereby the protein was repeatedly concentrated and diluted back to a buffer containing 20 mM Bis-tris, 300 mM NaCl, 1% glycerol, 1 mM tris(2-carboxyethyl)phosphine hydrochloride (TCEP-HCl), pD 5.5, prepared in 99.90% D_2_O (EURISO-TOP).

### Mass spectrometry

The mutations were verified using mass spectrometry. Accurate molecular masses were determined by liquid chromatography (LC)-coupled electrospray ionization time-of-flight mass spectrometry (ESI-TOF MS) in positive ion mode, using a Micromass Q-TOF 2 coupled with an ESI source. LC was performed using a Waters 2695 separation module with a Waters 2.1 mm × 10 mm reverse-phase MassPREP on-line desalting cartridge at +20 °C. Protein sequences were verified using peptide fingerprinting by in-gel trypsin proteolysis and matrix-assisted laser desorption/ionization time-of-flight mass spectrometry (MALDI-TOF MS) with a Bruker Ultra fleXtreme mass analyzer. The matrix used was α-cyano-4-hydroxy cinnamic acid (Bruker).

The dCNPcat perdeuteration degree was determined using ESI-TOF MS. Separate samples of pure CNPcat and dCNPcat were mixed with an excess of either H_2_O or D_2_O, and their masses were determined using a Waters Acquity Synapt G2 mass analyzer with a Z-Spray ESI source and compared to theoretical values. To determine the amount of exchanging protons/deuterons, the exchange reaction mixtures were incubated at ambient temperature, the reactions were quenched by adding trifluoroacetic acid to 0.1% at different timepoints, and measurements were carried out immediately. The reactions were considered complete, when the measured masses seized to change. For rapid measurements, protein solutions were directly injected into the ESI source and mass analyzer. The CNPcat total hydrogen amount was calculated using ProtParam^61^ and the amount of exchangeable protons using MS Tools^62^.

### Small-angle X-ray scattering

SAXS data were collected from samples concentrated up to 7–9 mg/ml in 20 mM Bis-tris, 300 mM NaCl, 10% glycerol, 1 mM TCEP-HCl, pH 5.5 on the ID14-3 BioSAXS beamline, European Synchrotron Radiation Facility (Grenoble, France) and the I911-4 Cassiopeia beamline, MAX-Lab (Lund, Sweden). Freshly prepared monomeric bovine serum albumin was used as a molecular weight standard. The data were processed and analyzed using BioXTAS RAW^63^ and ATSAS^64^. GNOM^65^ was used to calculate distance distribution functions.

### Circular dichroism spectroscopy

To compare the secondary structure content of the CNPcat variants, SRCD data were collected from 1 mg/ml samples in 10 mM sodium phosphate, pH 7.0 on the CD1 beamline at the ASTRID storage ring (ISA, Aarhus, Denmark) and on the UV-CD12 beamline at ANKA (KIT, Karlsruhe, Germany). The spectra were measured in 100-μm quartz cuvettes at +25 °C. Three scans of each sample from 280 to 170 nm were averaged, and the corresponding buffer spectrum was subtracted. The spectra were subjected to secondary structure deconvolution on DichroWeb^66^, using the CDSSTR algorithm and the SP175 reference database^67^,^68^.

Thermal stability of dCNPcat was analyzed using the Chirascan Plus spectropolarimeter (Applied Photophysics) as described^69^. The protein concentration was 0.25 mg/ml. The quartz cell had a pathlength of 0.5 mm, and the buffer consisted of 3 mM Bis-Tris (pH 5.5), 45 mM NaCl, 0.15% glycerol, and 0.15 mM TCEP-HCl. Spectra were measured continuously during heating from +20 to +90 °C, at a heating rate of 1 °C/min. The data were analyzed using Global 3^TM^ (Applied Photophysics).

### Enzyme activity and thermal stability

Enzyme activity assays were carried out as described earlier using BIO-TEK Instruments Inc. PowerWave X, Tecan Infinite M200, and M1000Pro spectrophotometers^38^,^48^. CNPcat catalyzes the conversion of 2′,3′-cNADP^+^ to NADP^+^, which is further converted to NAPDH by glucose-6-phosphate dehydrogenase in the presence of glucose-6-phosphate and MgCl_2_, and the absorbance at 340 nm is recorded as a function of time^48^. The substrate was 0.02 – 2 mM 2′,3′-cNADP^+^. The thermal stability of the mutants was assayed by exploiting the fluorescence intensity change of SYPRO Orange (Invitrogen) as described earlier^34^, including different ligands at 10 mM final concentration. The ligands included 2′,3′-cAMP, 2′-AMP, 3′-AMP, 5′-AMP, 2′,3′-cCMP, NADP^+^, ATP, and 2′,3′-cNADP^+^. Additionally, the enzyme activity and thermal stability of CNPcat and dCNPcat were assayed in both D_2_O and H_2_O. All activity and thermal shift assays were carried out in triplicate.

### Protein crystallization, data collection, structure determination, and refinement

CNPcat mutants were crystallized using the setup described earlier^34^. Sitting-drop vapor diffusion was performed against 85–100 μl reservoir solution containing 20–35% (w/v) PEG3350, PEG4000, or PEG6000 as precipitants and 50 mM Na acetate or 100 mM Na citrate, pH 3–5 as buffers at +4 °C and +20 °C. Protein solutions consisted of 250 μM (6 mg/ml) protein in 20 mM Bis-tris, 300 mM NaCl, 10% glycerol, 1 mM TCEP-HCl, pH 5.5. Co-crystallization experiments were used to incorporate ligands into the active site. Final concentrations of 5 or 10 mM ligand were added to the protein solutions. Ligands included 2′,3′-cAMP, the Sp epimer of 2′,3′-cyclic adenosine monophosphorothioate (2′,3′-Sp-cAMPS), 2′,3′-cNADP^+^, and 2′,5′-ADP. Each crystallization drop consisted of 0.5 μl protein and 0.5 μl reservoir solution. The crystallization conditions are shown in Supplementary Table S5. Both monoclinic and orthorhombic crystal plates grew even in the same drops. Before mounting, 1 μl of a cryoprotectant solution, consisting of reservoir solution supplemented with 23 – 25% PEG200 or PEG300, was added directly onto the drop.

Diffraction data were collected at 100 K on the synchrotron radiation beamlines X12, P13, and P14, EMBL/DESY (Hamburg, Germany) and I911-2, MAX-Lab (Lund, Sweden). Data were processed using XDS^70^. Phasing was done with molecular replacement using the mouse CNPcat (PDB ID 2xmi^38^) as the search model in Phaser^71^. Structure refinement was performed in phenix.refine^72^ and model building in Coot^73^. Apart from the highest-resolution structures, automatic optimization of the refinement weighting scheme was applied. The structures were validated and analyzed using DSSP^74^, MolProbity^75^, PyMOL, UCSF Chimera^76^, APBS^77^,^78^, and CCP4mg^79^. Data collection and refinement statistics are listed in Supplementary Table S1. Since all individual structure refinement procedures included both molecular replacement, building of solvent regions and ligands, addition of hydrogen atoms to riding positions, a number of cycles of manual rebuilding, and refinement until convergence, the test set of reflections was individually selected for each dataset. All discussed features were clearly visible in electron density maps, and model bias is not a concern. Recently, it was shown that refinement to convergence alone is enough to remove model bias^80^.

Crystallization of dCNPcat was based on conditions optimized for CNPcat^38^. The crystal used for data collection was grown using hanging-drop vapor diffusion on Nextal X plates at +8 °C. The well solution consisted of 50 mM acetate, 25% PEG3350, pD 3.5. Cryoprotection was done by adding 20% PEG200. The crystal was cryo-cooled in liquid nitrogen prior to data collection on beamline I911-3 (MAX-Lab). Processing, structure solution, and refinement were performed as above. Anisotropic B factors were refined, and deuterium atoms were added to their riding positions.

### PDB references

Catalytic domain of mouse 2′,3′-cyclic nucleotide 3′-phosphodiesterase, with mutations H230Q and H309Q, 4wbi

Catalytic domain of mouse 2′,3′-cyclic nucleotide 3′-phosphodiesterase, with mutation F235A, 4wbl

Catalytic domain of mouse 2′,3′-cyclic nucleotide 3′-phosphodiesterase, with mutation F235L, 4wc9

Catalytic domain of mouse 2′,3′-cyclic nucleotide 3′-phosphodiesterase, with mutation H230Q, complexed with citrate, 4wca

Catalytic domain of mouse 2′,3′-cyclic nucleotide 3′-phosphodiesterase, with mutation H309Q, 4wcb

Catalytic domain of mouse 2′,3′-cyclic nucleotide 3′-phosphodiesterase, with mutation P225G, 4wcc

Catalytic domain of mouse 2′,3′-cyclic nucleotide 3′-phosphodiesterase, with mutation P296G, complexed with 2′-AMP, 4wda

Catalytic domain of mouse 2′,3′-cyclic nucleotide 3′-phosphodiesterase, with mutation R307Q, complexed with 2′-AMP, 4wdb

Catalytic domain of mouse 2′,3′-cyclic nucleotide 3′-phosphodiesterase, with mutation T232A, complexed with citrate, 4wdd

Catalytic domain of mouse 2′,3′-cyclic nucleotide 3′-phosphodiesterase, with mutation T232A, complexed with 2′-AMP, 4wfr

Catalytic domain of mouse 2′,3′-cyclic nucleotide 3′-phosphodiesterase, with mutation T311A, 4wde

Catalytic domain of mouse 2′,3′-cyclic nucleotide 3′-phosphodiesterase, with mutation V321A, complexed with 2′,5′-ADP, 4wdf

Catalytic domain of mouse 2′,3′-cyclic nucleotide 3′-phosphodiesterase, with mutation V321A, complexed with 2′,5′-ADP, 4wdg

Catalytic domain of mouse 2′,3′-cyclic nucleotide 3′-phosphodiesterase, with mutation Y168A, 4wdh

Catalytic domain of mouse 2′,3′-cyclic nucleotide 3′-phosphodiesterase, with mutation Y168S, 4wex

Perdeuterated catalytic domain of mouse 2′,3′-cyclic nucleotide 3′-phosphodiesterase, 5ae0

## Additional Information

**How to cite this article**: Raasakka, A. *et al.* Determinants of ligand binding and catalytic activity in the myelin enzyme 2',3'-cyclic nucleotide 3'-phosphodiesterase. *Sci. Rep.* **5**, 16520; doi: 10.1038/srep16520 (2015).

## Supplementary Material

Supplementary Information

## Figures and Tables

**Table 1:** Enzyme activity and thermal stability.

variant	Phosphodiesterase activity (2′,3′-cNADP^+^ → 2′-NADP^+^)			T_m_
	k_cat_ (s^−1^)	K_M_ (μM)	k_cat_/K_M_(s^−1^ μM^−1^)	°C
Wild type*,**	940 ± 38	553 ± 46	1.70	58.0
H230Q**	24 ± 15	1055 ± 1130	0.02	55.0
H309Q**	–	–	–	52.7
H230S**	14 ± 14	1305 ± 2004	0.01	56.0
H309S**	21 ± 16	1192 ± 1468	0.02	57.3
H230Q & H309Q**	–	–	–	49.7
F235A	183 ± 15	6253 ± 798	0.03	53.0
F235L	221 ± 15	3788 ± 434	0.06	55.3
T232A	231 ± 136	39648 ± 25605	< 0.01	57.7
T311A	–	–	–	56.0
R307Q	1182 ± 205	2192 ± 548	0.54	59.0
V321A**	732 ± 33	1045 ± 77	0.70	59.5
Y168A	237 ± 7	1879 ± 122	0.13	53.7
Y168S	887 ± 98	1385 ± 235	0.64	53.7
P225G	1420 ± 60	483 ± 44	2.94	57.0
P296G	1332 ± 50	722 ± 51	1.73	60.0
**Protein, solvent**	**k_cat_ (s^**−**1^)**	**K_M_ (μM)**	**k_cat_/K_M_ (s^**−**1^ μM^**−**1^)**	**° C**
CNPcat, H_2_O*	807 ± 52	423 ± 76	1.91	60.0
CNPcat, D_2_O	273 ± 19	147 ± 38	1.85	61.0
dCNPcat, H_2_O	893 ± 57	382 ± 70	2.34	58.0
dCNPcat, D_2_O	326 ± 27	204 ± 57	1.59	59.0

All k_cat_ and K_M_ values are supplied with their respective standard errors. All T_m_ values were determined in the absence of ligands.

^*^CNPcat and wild type here refer to the same protein, but the measured values have been determined at different times and therefore slightly vary from each other. The magnitude of variation is small.

^**^The results have been discussed earlier (ref. 34) and are presented here for comparison.
