# Supplementary material for: Determinants of ligand binding and catalytic activity in the myelin enzyme 2′,3′-cyclic nucleotide 3′-phosphodiesterase
Source: Sci Rep. 2015 Nov 13;5:16520. doi: 10.1038/srep16520 (PMC4643303; doi:10.1038/srep16520)
Supplement: Supplementary Information [file srep16520-s1.pdf]

Determinants of ligand binding and catalytic activity in the myelin enzyme 2',3'-cyclic nucleotide 3'-phosphodiesterase

Supplementary Information

Arne Raasakka[1,2,3,4], Matti Myllykoski[1,2], Saara Laulumaa[1,2,4,5], Mari Lehtimäki[1], Michael Härtlein[6], Martine Moulin[6], Inari Kursula[1,3,4] & Petri Kursula[1,2,3,4,*]

[1]Faculty of Biochemistry and Molecular Medicine, University of Oulu, Oulu, Finland

[2]Biocenter Oulu, University of Oulu, Oulu, Finland

[3]Department of Biomedicine, University of Bergen, Bergen, Norway

[4]Helmholtz Centre for Infection Research at German Electron Synchrotron (DESY), Hamburg, Germany

[5]European Spallation Source (ESS), Lund, Sweden

[6]Institut Laue-Langevin (ILL), Grenoble, France

[*]Corresponding author: Petri Kursula, petri.kursula@biomed.uib.no

Supplementary Figure S1. SAXS & CD analysis of CNPcat variants and dCNPcat. In the case of all CNPcat variants, the obtained SAXS results appear similar, and the SAXS data are presented as an example for the R307Q mutant: (A) X-ray solution scattering data after background subtraction and Guinier analysis. (B) Full scattering data rearranged to a Kratky plot has a typical bell-shaped curve of a globular protein. The increase in $I \cdot s^2$ at high s-values indicates conformational flexibility. (C) Guinier fit with the determined R_g and I_0 values. (D) Distance distribution function with the determined D_{max} value and the used regularization parameter α. (E) SRCD data of all wild-type and mutant CNPcat variants. All proteins are folded, with little variation in overall curve shape. (F) Temperature scan using in-house CD for dCNPcat in D_2O shows a steep transition at 57 °C from mixed secondary structure content to temperature-induced β-aggregation, indicating thermal denaturation. The apparent thermal denaturation midpoint corroborates well with thermal shift assay data and is also close to the T_m of hydrogenated CNPcat.

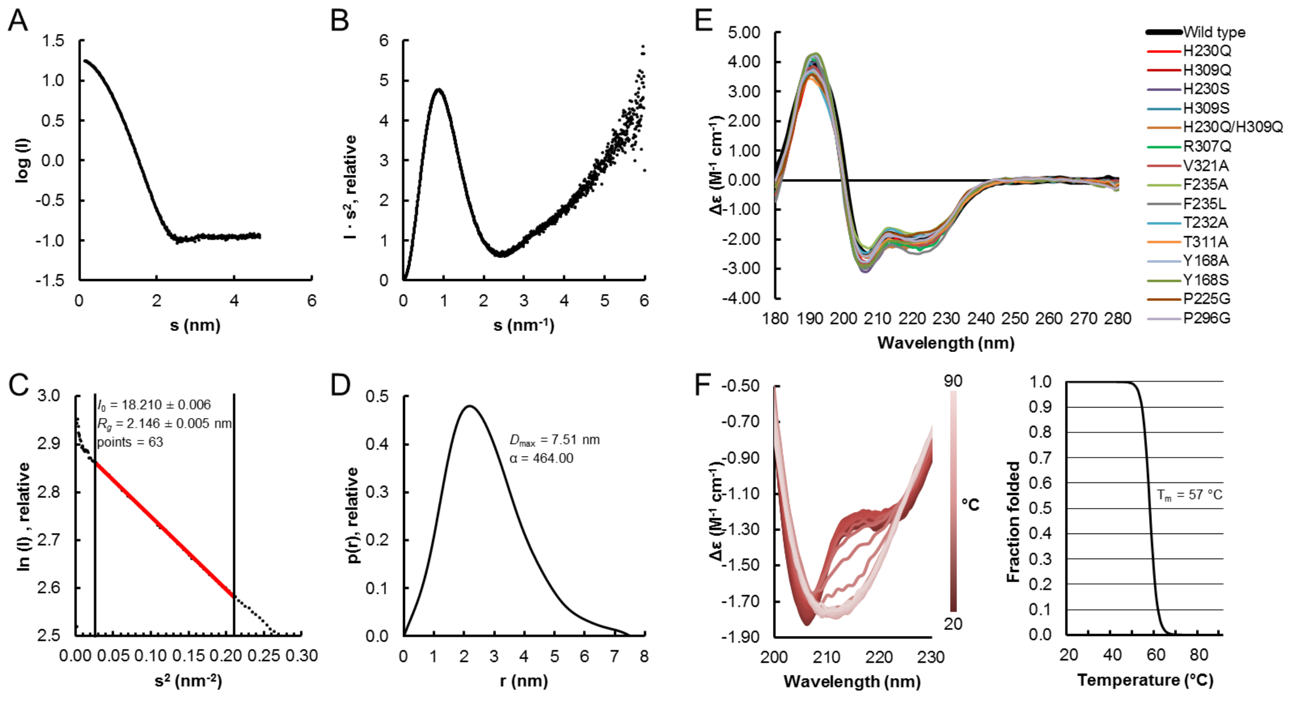

Supplementary Figure S2. Omit maps (F_o-F_c) for ligands within CNPcat mutant structures. (A) The electron density in the active site of T232A fits to a 2′-AMP product, indicating residual catalytic activity for this mutant. (B) The density within the active site of R307Q fits a 2′-AMP product in a mixed conformation. (C) Omit maps for V321A crystallized with 2′,5′-ADP in two different space groups. (D) The extra densities for active site-bound 2′-AMP in P225G and P296G reveal differences in ligand occupancy: in P225G, the product has a lower occupancy than for P296G. All omit maps are contoured at 3σ, with the exception of P225G contoured at 2σ.

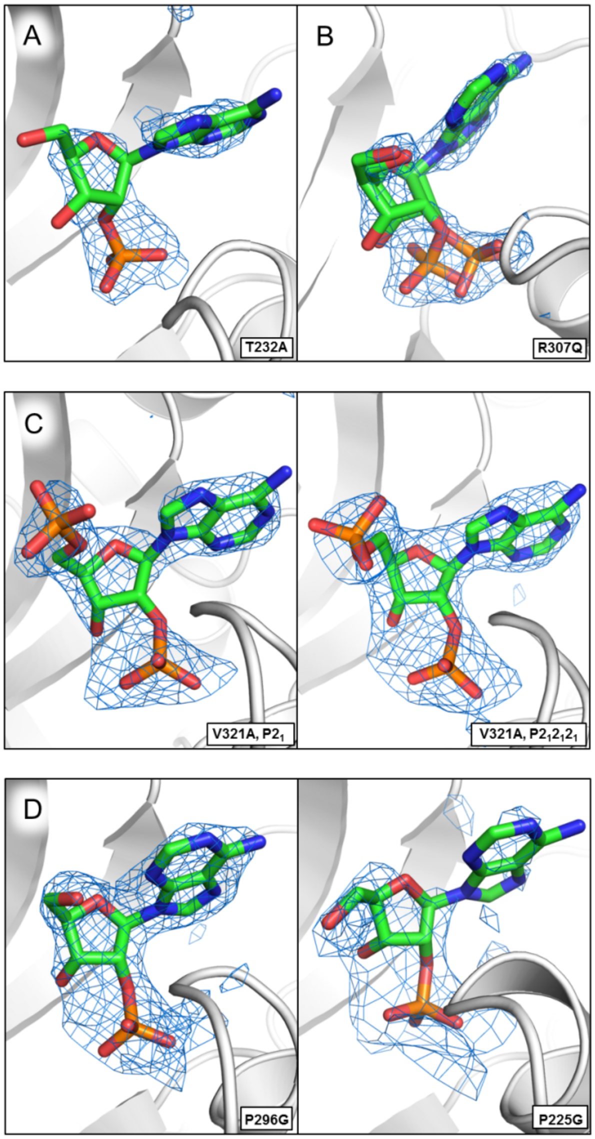

Supplementary Figure S3. CNPcat thermal stability in the presence of ligands. All T_m values were obtained based on the midpoint of rapid elevation of SYPRO Orange fluorescence as a function of temperature. Error margins have been calculated from triplicate values. The structures of the used ligands are shown below to clarify their differences.

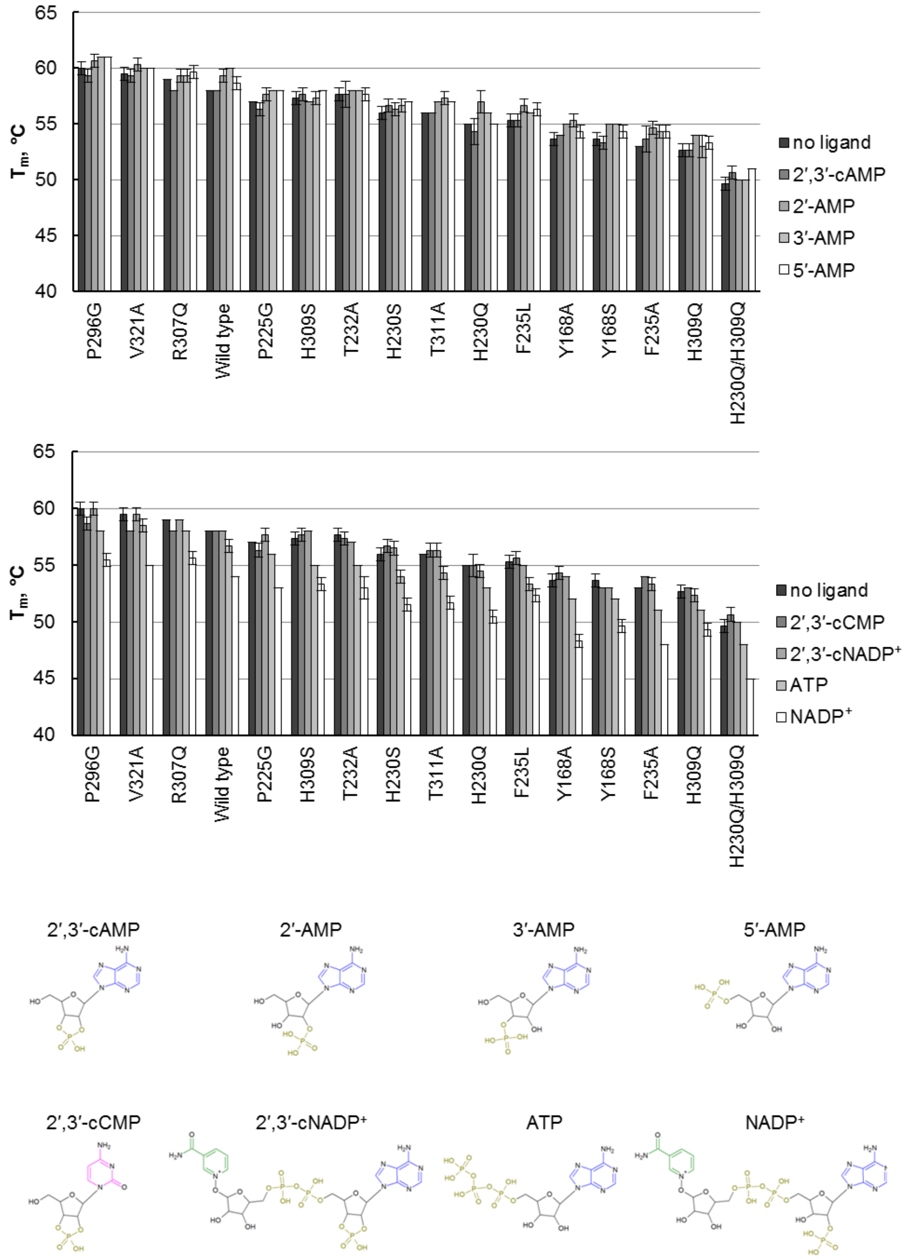

Supplementary Table S1. Data processing and structure refinement statistics. Data for the highest-resolution shell are in parentheses.

PDB entry	4wbi	4wbl	4wc9	4wca	4wcb	4wcc	4wda	4wdb	4wdd	4wfr	4wde	4wdf	4wdg	4wdh	4wex	5ae0
Mutation	H230Q & H309Q	F235A	F235L	H230Q	H309Q	P225G	P296G	R307Q	T232A	T232A	T311A	V321A	V321A	Y168A	Y168S	dCNP
Crystallization additive	2',3'-Sp-cAMPS	none	none	none	2',3'-cAMP	2',3'-cNADP	2',3'-cAMP	2',3'-cAMP	none	2',3'-cAMP	none	2',5'-ADP	2',5'-ADP	none	2',3'-cAMP	none
Active site ligand in structure	none	glycerol	none	citrate	none	none	2'-AMP	2'-AMP	citrate	2'-AMP	none	2',5'-ADP	2',5'-ADP	none	none	none
Wavelength (Å)	1.041	1.223	1.223	0.979	1.041	0.979	1.031	1.031	0.979	1.041	0.979	1.031	1.031	1.223	1.223	0.913
Resolution range (Å)	29 - 2.00 (2.07 - 2.00)	27 - 2.50 (2.59 - 2.50)	28 - 2.0 (2.07 - 2.00)	20 - 1.85 (1.92 - 1.85)	29 - 1.57 (1.63 - 1.57)	35 - 2.70 (2.80 - 2.70)	53 - 1.85 (1.90 - 1.85)	36 - 1.60 (1.64 - 1.60)	20 - 2.10 (2.18 - 2.10)	34 - 2.00 (2.07 - 2.00)	20 - 2.40 (2.49 - 2.40)	35 - 2.00 (2.05 - 2.00)	53 - 2.05 (2.10 - 2.05)	54 - 1.90 (1.97 - 1.90)	51 - 2.10 (2.17 - 2.10)	30-1.04 (1.07-1.04)
Space group	$P2_12_12_1$	$P2_1$	$P2_1$	$P2_12_12_1$	$P2_12_12_1$	$P2_12_12_1$	$P2_12_12_1$	$P2_12_12_1$	$P2_12_12_1$	$P2_1$	$P2_1$	$P2_1$	$P2_12_12_1$	$P2_1$	$P2_1$	$P2_1$
Unit cell a,b,c (Å) α,β,γ (°)	41.1 47.0 108.3 90 90 90	40.2 47.8 54.1 90 94.7 90	40.4 47.8 54.2 90 94.8 90	40.4 47.6 107.7 90 90 90	38.8 48.3 108.1 90 90 90	40.8 46.5 106.3 90 90 90	40.7 46.8 106.2 90 90 90	38.9 47.9 107.4 90 90 90	42.2 46.0 106.7 90 90 90	41.8 47.1 54.0 90 94.4 90	42.4 46.4 107.5 90 93.9 90	41.1 47.0 53.7 90 95.7 90	41.3 46.9 106.9 90 90 90	39.3 48.3 54.0 90 94.5 90	38.2 48.2 51.2 90 95.6 90	39.65 47.56 51.22 90 97.51 90
Unique reflections	14675 (1420)	7167 (707)	13840 (1359)	18374 (1795)	29128 (2885)	5828 (559)	17983 (1307)	27315 (1991)	12659 (1238)	13867 (1349)	16409 (1523)	13871 (1034)	13665 (991)	14961 (1205)	10929 (1044)	90347 (6610)
Multiplicity	6.0 (5.9)	3.6 (3.6)	3.2 (2.9)	6.4 (4.2)	6.9 (6.2)	4.4 (4.1)	6.9 (7.0)	7.1 (6.8)	5.7 (5.6)	3.3 (3.2)	3.6 (3.1)	3.6 (3.6)	6.6 (6.7)	2.9 (1.9)	3.6 (3.1)	3.7 (2.9)
Completeness (%)	99.0 (97.8)	99.7 (99.9)	98.3 (97.1)	99.9 (99.3)	99.9 (99.6)	98.1 (97.6)	99.8 (99.9)	100.0 (100.0)	99.9 (100.0)	97.1 (96.1)	99.0 (91.5)	99.4 (99.9)	99.8 (99.9)	93.1 (75.6)	99.5 (97.2)	99.7 (99.1)
<I/σ(I)>	14.6 (2.4)	9.0 (1.9)	8.5 (2.1)	15.4 (1.9)	18.4 (2.2)	17.0 (1.9)	12.8 (1.2)	18.8 (1.0)	9.8 (1.8)	9.4 (2.7)	7.0 (1.3)	9.3 (1.5)	8.7 (1.1)	9.2 (1.2)	8.5 (2.0)	9.6 (1.1)
Wilson B-factor ($Å^2$)	32.6	30.6	25.9	20.8	16.0	56.1	36.0	26.2	32.6	19.3	29.4	36.4	43.5	23.8	24.7	12.2
R_{merge}	0.074 (0.758)	0.200 (0.805)	0.088 (0.598)	0.086 (0.697)	0.071 (0.914)	0.070 (0.704)	0.076 (1.583)	0.052 (1.869)	0.142 (0.906)	0.114 (0.459)	0.187 (0.930)	0.084 (0.942)	0.137 (1.651)	0.072 (0.708)	0.110 (0.650)	0.074 (1.261)
$CC_{1/2}$	0.999 (0.749)	0.882 (0.616)	0.992 (0.77)	0.999 (0.686)	0.999 (0.764)	0.998 (0.610)	0.999 (0.639)	1 (0.344)	0.997 (0.514)	0.993 (0.617)	0.985 (0.472)	0.996 (0.381)	0.995 (0.497)	0.996 (0.749)	0.995 (0.747)	0.997 (0.455)
R_{work}	0.201	0.195	0.224	0.163	0.159	0.222	0.218	0.165	0.213	0.216	0.249	0.204	0.236	0.217	0.199	0.1270
R_{free}	0.229	0.265	0.271	0.208	0.204	0.268	0.249	0.202	0.271	0.267	0.299	0.259	0.292	0.259	0.239	0.1563
RMSD(bonds) (Å)	0.007	0.003	0.003	0.008	0.007	0.010	0.004	0.017	0.003	0.003	0.002	0.011	0.010	0.003	0.003	0.019
RMSD(angles) (°)	1.0	0.6	0.7	1.1	1.1	0.9	0.8	1.8	0.6	0.8	0.6	1.3	1.0	0.7	0.7	1.6
Ramachandran favored (%)	99	99	99	99	99	97	98	96	97	98	98	99	97	97	99	97
Ramachandran outliers (%)	0	0	0	0	0	0	0.5	0.5	0.5	0	0	0	0	0	0	0

Supplementary Table S2. Mass spectrometric identity analysis of CNPcat mutants.

	Accurate molecular weight			Peptide fingerprinting	
**Protein**	**Theoretical mass (Da)**	**Iterated mass (Da)**	**Difference (Da)**	**Sequence coverage (%)**	**Mutation covered**
Wild type	24259.9	24262	2.1	71.5	–
H230Q	24250.9	24253	2.1	90.5	Yes
H309Q	24250.9	24253	2.1	90.5	Yes
H230S	24209.8	24212	2.2	91.9	Yes
H309S	24209.8	24212	2.2	90.1	Yes
H230Q/H309Q	24241.9	24244	2.1	94.1	Yes
R307Q*	24231.8	24234	2.2	51.5	No
V321A	24231.8	24234	2.2	92.3	Yes
F235A	24183.8	24186	2.2	80.5	Yes
F235L**	24225.9	24229	3.1	84.2	No
T232A	24229.9	24232	2.1	91.0	Yes
T311A	24229.9	24232	2.1	90.5	Yes
Y168A	24167.8	24171	3.2	91.9	Yes
Y168S	24183.8	24186	2.2	86.0	Yes
P225G	24219.8	24221	1.2	79.6	Yes
P296G***	24219.8	24221	1.2	89.2	Yes
Myoglobin	16951.5	16953	1.5	–	–

* R307 is a trypsination site, and therefore, the peptides covering the mutation are absent. The desired mutation is visible in the crystal structure (PDB ID 4wdb)

** The peptide covering the mutation was absent from this mass spectrum. The desired mutation is visible in the crystal structure (PDB ID 4wc9)

*** This mutation resides in a disordered loop and is not visible in the crystal structure (PDB ID 4wda)

Supplementary Table S3. Hydrogen-deuterium exchange analysis of CNPcat and dCNPcat.

	Accurate molecular weight			
**Protein**	**Theoretical mass (Da)**	**Determined mass (Da)**	**Difference (Da)**	**Exchanged***
CNPcat, H_2O	24259.9	24259.5	0.4	–
CNPcat, D_2O	24659.9	24576.5	83.4	317 (79%)
dCNPcat, D_2O	26012.1	25976.0	36.1	–
dCNPcat, H_2O	25576.0**	25599.5	-23.5	377 (94%)**

* Exchanged protons/deuterons are the differences compared to the measured proteins in their native solvent. Percentage of exchange compared to 401 total exchangeable protons in brackets

** The theoretical mass has been calculated based on the measured molecular weight of dCNPcat in D_2O

Supplementary Table S4. CNPcat variant SAXS results and CD deconvolutions.

	SAXS results					Deconvoluted CD spectra	
**Protein**	**Beamline**	**I(0)**	**M_r (Da)**	**R_g (nm)**	**D_{max} (nm)**	**% helix**	**% sheet**
Wild type*	ID14-3 / I911-4	18.6 / 2.1	19 / 21	2.2 / 2.0	7.6 / 7.1	19 (30)	29 (29)
H230Q	ID14-3	17.8	18	2.1	7.4	19	29
H309Q	ID14-3	17.7	18	2.1	7.3	19	29
H230S	ID14-3	18.1	18	2.1	7.4	19	28
H309S	ID14-3	17.8	18	2.1	7.3	19	28
H230Q & H309Q	ID14-3	17.4	18	2.1	7.3	19	29
F235A	I911-4	2.0	21	2.0	7.4	17	33
F235L	I911-4	2.0	21	2.0	7.5	23	26
T232A	I911-4	2.1	22	2.2	7.8	16	31
T311A	I911-4	2.1	21	2.1	7.2	19	29
R307Q	ID14-3	18.2	18	2.2	7.5	21	27
V321A	ID14-3	18.0	18	2.1	7.5	19	28
Y168A	I911-4	2.3	26	2.5	7.6	17	30
Y168S	I911-4	2.5	26	2.3	7.7	19	30
P225G	ID14-3	18.9	19	2.2	7.7	17	30
P296G	ID14-3	17.6	18	2.1	7.3	18	30
BSA	ID14-3 / I911-4	65.3 / 6.5	66.7	–	–	–	–

* The secondary structure percentages in brackets have been calculated from the crystal structure (PDB ID 2xmi, *ref. 38*) using *DSSP*.

Supplementary Table S5. Crystallization conditions for individual structures.

PDB entry	Variant	Buffer	Precipitant	Temp (°C)	Ligand additive	Cryoprotection
4wca	H230Q	100 mM tri-sodium citrate, pH 3.5	25% PEG3350	4	–	50 mM tri-sodium citrate, pH 4, 23 % PEG4000, 23% PEG200
4wcb	H309Q	50 mM sodium acetate, pH 3.0	30% PEG4000	4	10 mM 2',3'-cAMP	50 mM sodium acetate, pH 4, 23 % PEG4000, 23% PEG300
4wbi	H230Q/H309Q	50 mM tri-sodium citrate, pH 3.4	30% PEG 6000	4	5 mM 2',3'-Sp-cAMPS	50 mM tri-sodium citrate, 30% PEG6000, 13% PEG200
4wdd	T232A	50 mM tri-sodium citrate, pH 3.4	30% PEG6000	20	–	50 mM tri-sodium citrate, pH 4, 23 % PEG4000, 23% PEG200
4wfr	T232A	50 mM sodium acetate, pH 4.0	25% PEG6000	4	10 mM 2',3'-cAMP	50 mM sodium acetate, pH 4, 23 % PEG4000, 23% PEG300
4wde	T311A	50 mM tri-sodium citrate, pH 3.4	30% PEG6000	20	–	50 mM tri-sodium citrate, pH 4, 23 % PEG4000, 23% PEG200
4wdf	V321A	50 mM sodium acetate, pH 3.0	35% PEG6000	4	5 mM 2',5'-ADP	100% PEG200
4wdg	V321A	50 mM sodium acetate, pH 3.0	35% PEG6000	4	5 mM 2',5'-ADP	100% PEG200
4wdb	R307Q	50 mM sodium acetate, pH 4.0	35% PEG4000	4	10 mM 2',3'-cAMP	100% PEG200
4wbl	F235A	50 mM sodium acetate, pH 3.4	30% PEG6000	4	–	20% PEG200
4wc9	F235L	50 mM sodium acetate, pH 3.7	25% PEG6000	20	–	20% PEG200
4wdh	Y168A	50 mM sodium acetate, pH 3.0	30% PEG6000	20	–	20% PEG200
4wex	Y168S	50 mM sodium acetate, pH 3.4	30% PEG4000	20	10 mM 2',3'-cAMP	20% PEG200
4wcc	P225G	50 mM tri-sodium citrate, pH 3.1	30% PEG6000	4	10 mM $cNADP^+$	50 mM tri-sodium citrate, 30% PEG6000, 13% PEG200
4wda	P296G	50 mM sodium acetate, pH 3.0	35% PEG6000	4	10 mM 2',3'-cAMP	100% PEG200
5ae0	dCNPcat*	50 mM tri-sodium acetate, pD 3.5	25 % PEG3350	8	–	20% PEG200

* Crystallization was carried out with D_2O as solvent
